# Biomechanical performance of the novel assembled uncovertebral joint fusion cage in single-level anterior cervical discectomy and fusion: A finite element analysis

**DOI:** 10.3389/fbioe.2023.931202

**Published:** 2023-03-08

**Authors:** Xiang Zhang, Yi Yang, Yi-Wei Shen, Ke-Rui Zhang, Li-Tai Ma, Chen Ding, Bei-Yu Wang, Yang Meng, Hao Liu

**Affiliations:** Department of Orthopedics, Orthopedic Research Institute, West China Hospital, Sichuan University, Chengdu, China

**Keywords:** finite element analysis, anterior cervical discectomy and fusion, assembled uncovertebral joint fusion cage, zero-P interbody fusion, bone fusion

## Abstract

**Introduction:** Anterior cervical discectomy and fusion (ACDF) is widely accepted as the gold standard surgical procedure for treating cervical radiculopathy and myelopathy. However, there is concern about the low fusion rate in the early period after ACDF surgery using the Zero-P fusion cage. We creatively designed an assembled uncoupled joint fusion device to improve the fusion rate and solve the implantation difficulties. This study aimed to assess the biomechanical performance of the assembled uncovertebral joint fusion cage in single-level ACDF and compare it with the Zero-P device.

**Methods:** A three-dimensional finite element (FE) of a healthy cervical spine (C2−C7) was constructed and validated. In the one-level surgery model, either an assembled uncovertebral joint fusion cage or a zero-profile device was implanted at the C5–C6 segment of the model. A pure moment of 1.0 Nm combined with a follower load of 75 N was imposed at C2 to determine flexion, extension, lateral bending, and axial rotation. The segmental range of motion (ROM), facet contact force (FCF), maximum intradiscal pressure (IDP), and screw−bone stress were determined and compared with those of the zero-profile device.

**Results:** The results showed that the ROMs of the fused levels in both models were nearly zero, while the motions of the unfused segments were unevenly increased. The FCF at adjacent segments in the assembled uncovertebral joint fusion cage group was less than that that of the Zero-P group. The IDP at the adjacent segments and screw–bone stress were slightly higher in the assembled uncovertebral joint fusion cage group than in those of the Zero-P group. Stress on the cage was mainly concentrated on both sides of the wings, reaching 13.4–20.4 Mpa in the assembled uncovertebral joint fusion cage group.

**Conclusion:** The assembled uncovertebral joint fusion cage provided strong immobilization, similar to the Zero-P device. When compared with the Zero-P group, the assembled uncovertebral joint fusion cage achieved similar resultant values regarding FCF, IDP, and screw–bone stress. Moreover, the assembled uncovertebral joint fusion cage effectively achieved early bone formation and fusion, probably due to proper stress distributions in the wings of both sides.

## Introduction

Anterior cervical discectomy and fusion (ACDF) is considered the gold standard surgical treatment for patients with cervical disc disease who have failed conservative treatments (Bohlman et al., 1993; Emery et al., 1998). Since its introduction by Cloward(1958) and Smith and Robinson (1958), the surgical technique has been continuously modified to improve fusion rates and clinical outcomes by optimizing the implant design and changing its materials (Hacker, 2000). Solid bone fusion after ACDF is one of the key indicators of achieving the expected clinical outcomes. As one of the most recommended fusion devices for ACDF, Zero-P devices use an integrated and low-profile plate design to reduce dysphagia rates and other plate-associated complications while maintaining satisfactory clinical outcomes (Barbagallo et al., 2013; Vanek et al., 2013; Chen et al., 2015). Previous studies have suggested that the fusion rates at 3 and 6 months after ACDF with Zero-P implant were 19.1% and 74.5%, respectively (He et al., 2020; Abudouaini et al., 2021a), but it did not achieve clinicians’ desired results of a 90% fusion rate as early as possible (Vanek et al., 2013).

The uncovertebral joint, known as the Luschka joint, is a unique anatomical structure of the cervical spine. It is located on either side of the C3–C7 vertebral body, is formed by the anastomosis of the uncinate process on the posterolateral side of the lower vertebral body with the lower slope of the upper vertebral body, and has been shown to play an important role in limiting cervical lateral orientation and movement and maintaining cervical stability (Hartman, 2014). In our clinical practice, obvious bony fusion was often detected in the uncovertebral joint area during anterior intervertebral space release surgery on patients with old cervical fractures and dislocations. Additionally, heterotopic ossification was most significantly distributed at the uncovertebral joint during long-term follow-up after artificial cervical disc replacement (Tian et al., 2016). Furthermore, our previous clinical studies showed that the application of Zero-profile anchoring spacers (Zero-P, Johnson & Johnson) in ACDF with bone grafting in the uncovertebral joint area is safe and effective, with fusion rates of 16.7%, 63%, and 98.1% at 3, 6, and 12 months after the surgery when compared to control fusion rates of 2.5%, 33.3%, and 88.9%, respectively, indicating the great potential of accelerating fusion and improving fusion capacity (Liu. et al., 2020). In our prospective, randomized, controlled trial study, the fusion rate in the uncovertebral joint fusion group was found to be significantly higher than in the traditional interbody fusion group at 3 and 6 months after operation (3 months: 70% *vs* 10%, *p* < 0.0001; 6 months: 95% *vs* 65%, *p* = 0.0177, respectively) (Hao et al., 2022).

Therefore, we postulated that uncovertebral joint fusion might have potential advantages in cervical spine interbody fusion and designed a novel uncovertebral joint fusion cage. In our previous goat model research (Shen et al., 2021), 75.0% (9/12) of the goats in the uncovertebral joint fusion cage group were evaluated as achieving fusion at 12 weeks when compared to 41.7% (5/12) in the non-profile cage group. Additionally, the fusion grading scores of the uncovertebral joint fusion cage group were significantly higher than those of the non-profile cage group, both at 12 and 24 weeks (*p* < 0.05), without increasing serious complications during the 6 months of follow-up. Furthermore, in the goat experiment, initial stability biomechanical tests showed that the uncovertebral joint fusion cage had slightly better stability than the Zero-P device in the right and left lateral flexion and axial rotation and comparable stability to the Zero-P device in anterior flexion and posterior extension (Yang. et al., 2019). However, the existing uncovertebral joint fusion cage cannot be used to perform both inter–end plate bone grafting and uncinate joint bone grafting.

Due to the differences in the physiological state and osteogenesis ability of different patients, a single bone grafting area limits the improvement of fusion efficiency to a certain extent. Also, implantation difficulties hinder further development of the uncovertebral joint fusion cage. Therefore, we designed the assembled uncovertebral joint fusion cage to make the surgery easier. A double bone grafting path combining inter–end plate bone grafting and uncovertebral joint bone grafting was designed, which not only ensures a good biomechanical environment for osteogenesis but also combines the advantages of the two fusion ideas to make bone grafting safer and more effective. Specifically, we designed an assembled uncovertebral joint fusion cage comprising an intervertebral body support and uncovertebral joint fusion components on either side. The uncovertebral joint fusion components had upper and lower penetrating bone graft cavities, and the intervertebral support body or uncovertebral joint fusion components had an anatomical surface pattern. We printed the bone graft material or artificially filled and compacted it into the bone graft area before surgery, eliminating the need to implant decompressed bone or artificial bone particles into the uncovertebral joint fusion area through surgical forceps, thus reducing the risk of complications caused by dislodged bone graft particles, shortening surgery time, and making the operation easier. In addition, because of the split design, we could use 3D printing technology to individually print the intervertebral support body and the uncovertebral joint fusion components in reference to previous studies which focused on 3D-printed discs or fusion devices (Serra et al., 2016; Basgul et al., 2020; Li et al., 2021; Zhu et al., 2021) to achieve a good fit between the intervertebral support body, the uncovertebral joint fusion components, and the patient’s vertebral space, thereby reducing the difficulty of surgical operation and improving the prosthesis–end plate fit.

However, a comprehensive finite element model has not yet been established to analyze its mechanical state. To the best of our knowledge, this is the first study to assess the biomechanical performance of the assembled uncovertebral joint fusion cage in single-level ACDF and compare it with the Zero-P device.

## Materials and methods

### Development of FE intact cervical spine model

A non-linear three-dimensional finite element (FE) model of the cervical spine segments (C2−C7) was developed and validated in our previous study (Rong et al., 2017). The model was constructed on the basis of computed tomography (CT) images from a young male volunteer without cervical degeneration (28 years, 165 cm, 65 kg), with a resolution of 0.75 mm and an interval of 0.69 mm from a CT scanner (SOMATOM Definition AS+, Siemens, Germany).

### Generation of cervical spine model and instrument

The CT scans were imported into the Mimics 19.0 (Materialize Inc., Leuven, Belgium) software to reconstruct the geometric structure of the C2−C7 cervical vertebrae. The corresponding tissues were distinguished according to CT grayscale and exported to STL or Cloud point cloud format. The intervertebral disc geometries were constructed by filling the intervertebral space and connecting the adjacent vertebral bodies. Next, a preliminary geometric model was established, followed by denoising, paving, and smoothing to optimize the geometric structure of the model with the CATIA V5 R21 (Dassault Systèmes Corporation, Velizy-Villacoublay Cedex, France) and importing it into HyperMesh 12.0 (Altair, Troy, MI, United States) to prepare mesh division, such as the cervical spine, intervertebral disc, ligament, and other structural mesh. Last, the boundary conditions of the prepared model were set using ABAQUS 6.9.1 (Dassault Systèmes Corporation).

**FIGURE 1:**
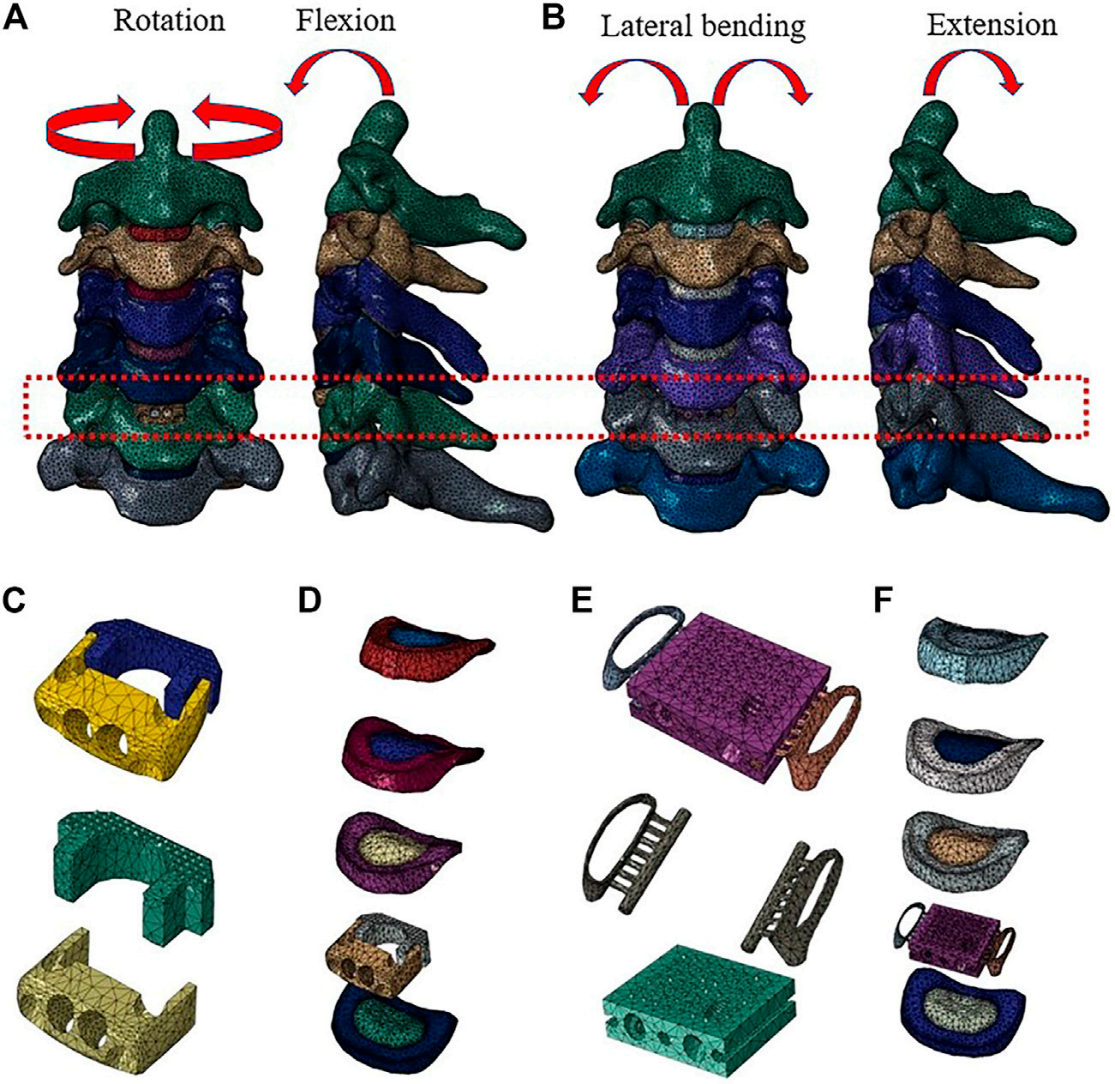
Finite element model of the C2–C7 cervical spine: **(A)** front view and sagittal view of the implanted Zero-P model, and **(B)** front view and sagittal view of the implanted assembled uncovertebral joint fusion cage model. **(C)** The Zero-P device (screws are not shown). **(D)** Intact intervertebral disk and the Zero-P device of the implanted Zero-P model. **(E)** The assembled uncovertebral joint fusion cage was composed of a body part, two wings, and screws (screws are not shown). **(F)** Intact intervertebral disk and the assembled uncovertebral joint fusion cage of the implanted assembled uncovertebral joint fusion cage model.

The cancellous bone regions of the vertebrae were set as solid elements. A 0.4-mm-thick shell consisting of the cortical bone and end plates covered the cancellous bone. The intervertebral disc was divided into the annulus fibrosus and nucleus pulposus with a volume ratio of 6:4. Annulus fibers surrounded the ground substance with an inclination to the transverse plane between 15° and 30,° accounting for approximately 19% of the entire annulus fibrosus volume (Denozière and Ku, 2006; Panzer and Cronin, 2009). The facet joint space was 0.5 mm and was covered by a cartilage layer with non-linear surface-to-surface contact. The ligamentous complex, which includes the anterior longitudinal ligament (ALL), posterior longitudinal ligament (PLL), ligamentum flavum, interspinous ligament, and capsular ligament, was developed using tension-only rod elements and was attached to the corresponding vertebrae. The Zero-P system (Synthes, Oberdorf, Switzerland), composed of the zero-profile titanium plate, polyetheretherketone (PEEK) cage, and two self-tapping screws in opposite directions, was adopted in this study. The primary dimensions (width, length, and height) were 13.6, 17.5, and 5 mm, respectively. The self-tapping screws were 16 mm long. Additionally, convergence analysis was performed to ensure that the maximum changes in the strain energy were <3% and showed that when the element size was between 0.1 and 1, the error stabilized at a minimum value, i.e., less than 3%, which is concordant with previous studies (Jones and Wilcox, 2008; Ayturk and Puttlitz, 2011; Zhang et al., 2022b). The material properties of the bone graft and newly formed bone were set as the cortical bone (Figure 1). The material properties and mesh types are listed in Table 1 (Denozière and Ku, 2006; Lee et al., 2011; Mo et al., 2017; Rong et al., 2017). The number of nodes and elements of the cervical spine model are shown in Table 2.

**TABLE 1:** Material properties and mesh types of the cervical finite element model.

Component	Young’s modulus (MPa)	Poisson’s ratio	Element type	Cross sections (mm^2^)
Cortical bone	12,000	0.29	C3D4	—
Cancellous bone	450	0.29	C3D4	—
Nucleus pulpous	1.0	0.49	C3D4	—
Facet joint cartilage	10.4	0.4	C3D4	—
Annulus fibers	110	0.3	T3D2	—
Anterior longitudinal ligament	10	0.3	T3D2	6.0
Posterior longitudinal ligament	10	0.3	T3D2	5.0
Capsular ligament	10	0.3	T3D2	46.0
Interspinous ligament	1.5	0.3	T3D2	10.0
Supraspinous ligament	1.5	0.3	T3D2	5.0
Ligamentum flavum	1.5	0.3	T3D2	5.0
Cage (titanium)	1,10,000	0.3	C3D4	—
Screws	1,10,000	0.3	C3D4	—
Cage (PEEK)	3,600	0.3	C3D4	—

Note: C3D4, tetrahedron; T3D2, truss, tension-only.

**TABLE 2:** Number of elements and nodes for the cervical spine model.

	Element	Node
C2	63,892	96,991
C3	62,239	95,895
C4	60,707	93,960
C5	68,221	1,07,543
C6	59,809	94,578
C7	65,885	1,03,284
C2/3	11,400	20,170
C3/4	7,621	14,121
C4/5	9,818	17,721
C6/7	11,101	20,026
Anterior longitudinal ligament	135	136
Posterior longitudinal ligament	158	159
Capsular ligament	150	200
Ligamentum flavum	137	138
Interspinous ligament	92	98
Supraspinous ligament	280	290

### Boundary conditions

A tie connection was assigned between the intervertebral discs and adjacent vertebral bodies and between the insertion of ligaments to the bone. The facet joint was built as a non-linear three-dimensional contact problem using surface-to-surface elements. Frictionless contact was defined between the articular surfaces of the facet joints (Panzer and Cronin, 2009; Rong et al., 2017). The cancellous bone that filled the central cavity of the cage was defined as frictionless (Completo et al., 2015). A non-bonded contact was applied between the cage’s supra- and infra-adjacent surfaces and the relevant vertebral surfaces with a contact friction coefficient of 0.3 (Galbusera et al., 2008). The graft–vertebrae and screw–vertebrae interfaces were defined as tie constraints to simulate rigid fusion and sufficient osseointegration. To simplify the model, shared nodes at the screw–plate interfaces were used, thus preventing relative motion between the components. The implant interfaces of the artificial cervical disc were defined as surface-to-surface sliding contact with a fraction coefficient of 0.07 (Li et al., 2018).

### Biomechanical testing

The FE model of intact C2−C7 segments was fixed at the inferior end plate of C7. Follower loads of 75 N were used to simulate muscle force and head weight. A 1.0-N/m moment and 75-N follower load were applied to the odontoid of the C2 vertebrae to produce flexion, extension, lateral bending, and axial rotation (Rong et al., 2017; Wu et al., 2019; Wo et al., 2021; Lu et al., 2022). The ALL, PLL, nucleus pulposus, and annulus fibrosus were resected at C5/6, while the bilateral structures, such as uncinate processes, were preserved according to real surgical procedures. The range of motion (ROM) was defined as the rotation from the neutral position to the end position with a 1.0-N/m load. The ROM for each level was calculated on the basis of the relative motions of the markers of each vertebra in each motion mode (Panjabi et al., 2001).

According to the hybrid control proposed by Panjabi et al. (2001), the corresponding movement angles of all directions in the intact cervical model were applied to the ACDF surgical constructs. The ROM of each segment of the intact cervical spine model under all moments was compared with previously published data to validate the model. Based on our previous study and literature data, we chose the C5/6 level as the implanted level because it is the most frequently involved level in clinical practice (Bisson et al., 2011; Wang et al., 2013; Qizhi et al., 2016; Shi et al., 2016; Wu et al., 2017).

## Results

### Validation of the intact cervical spine model

**FIGURE 2:**
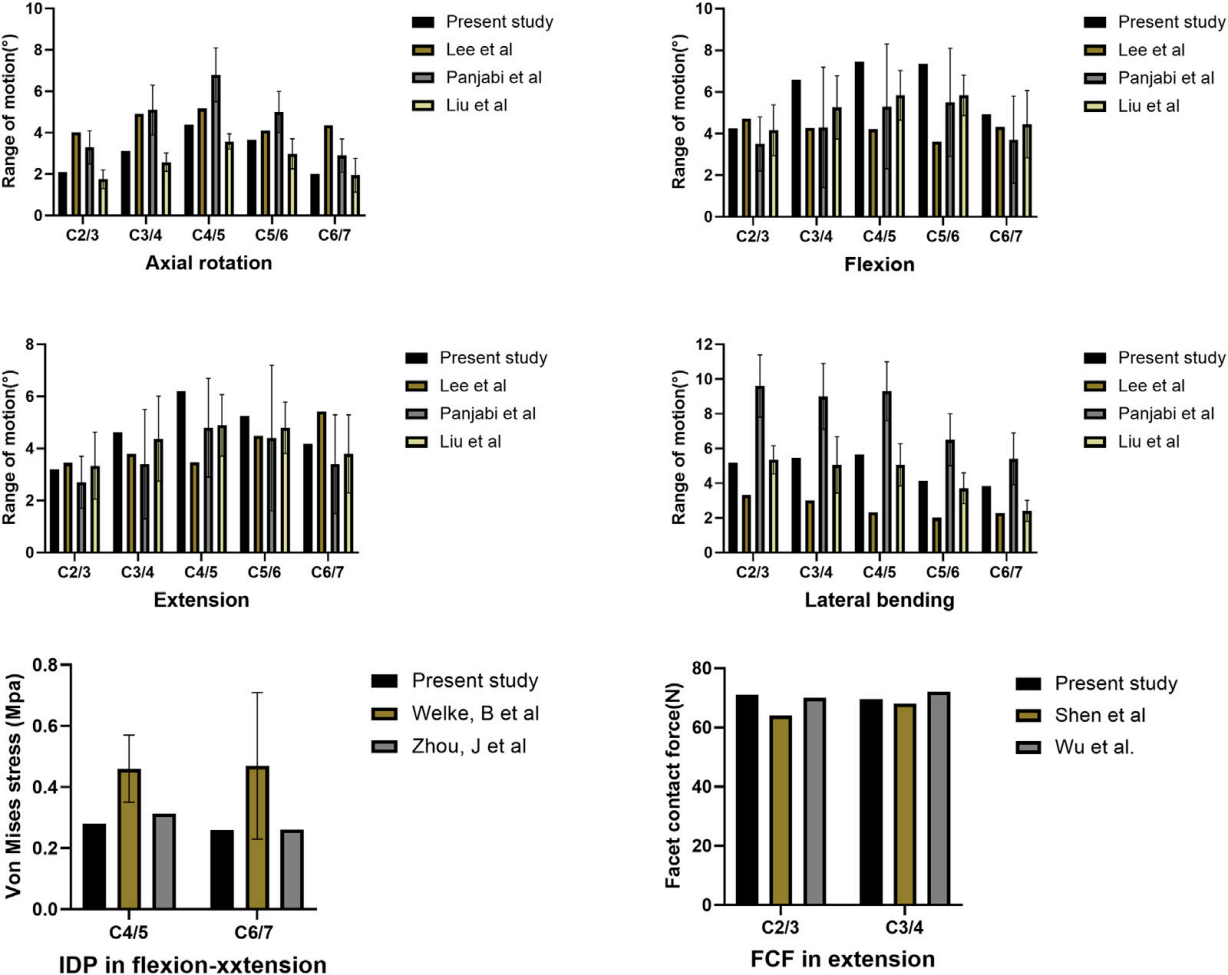
Comparison of the predicted ranges of motion (ROMs), intradiscal pressure (IDP), and facet contact force (FCF) with published literature.

As shown in Figure 2, the predicted segmental ROM of the present intact cervical spine model was within the standard deviation of the previous experimental data (Panjabi et al., 2001; Lee et al., 2016; Liu et al., 2016). The maximal intradiscal pressure of adjacent levels was consistent with *in vitro* experiments and previous finite element results (Welke et al., 2016; Zhou et al., 2021), and the facet contact force (FCF) of the model was also in agreement with the literature (Wu et al., 2019; Shen et al., 2022). All indicated that the present model was reliable in representing a healthy individual and could be used for further experiments.

### Range of motion

**FIGURE 3:**
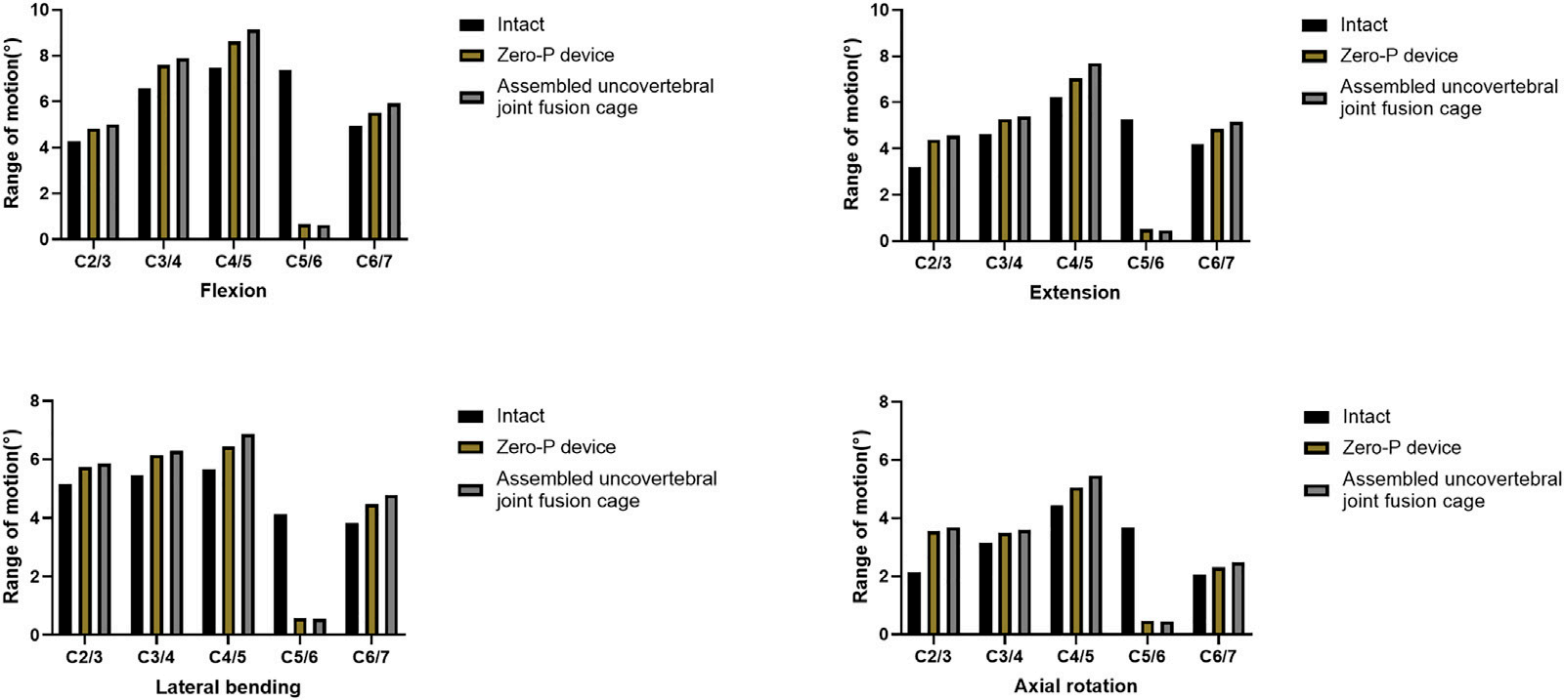
Range of motion (ROM) of three-dimensional finite element models of one-level anterior cervical discectomy and fusion using either the assembled uncovertebral joint fusion cage or Zero-P device.

The ROMs of each segment during flexion, extension, lateral bending, and axial rotation are shown in Figure 3. For all motions, the ROMs of the fused levels in the assembled uncovertebral joint fusion cage and Zero-P device models were nearly zero, and the ROMs of the unfused levels in all models were increased by 10.4%–73.2% for all motions when compared with the intact model. The unfused levels that exhibited increased motions fulfilled a compensatory function to maintain normal movement.

### Facet contact force

**FIGURE 4:**
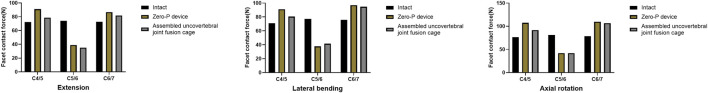
Facet contact force at the surgical and adjacent levels in extension, lateral bending, and axial rotation.

Under the extension moment, FCF tended to decrease by 52.6% and 47.4% at the fused levels in the assembled uncovertebral joint fusion cage and Zero-P device group, respectively. When compared to the intact model, the maximum increase in the facet joint force in the superior adjacent and inferior adjacent segments of the assembled uncovertebral joint fusion cage group was 9.0% and 12.7% under the extension moment, respectively. Additionally, under the extension moment, the facet joint force in the Zero-P device group increased by 26.0% and 19.3% in the superior adjacent and inferior adjacent segments, respectively, when compared with the intact model (Figure 4).

### Intradiscal pressure

**FIGURE 5:**
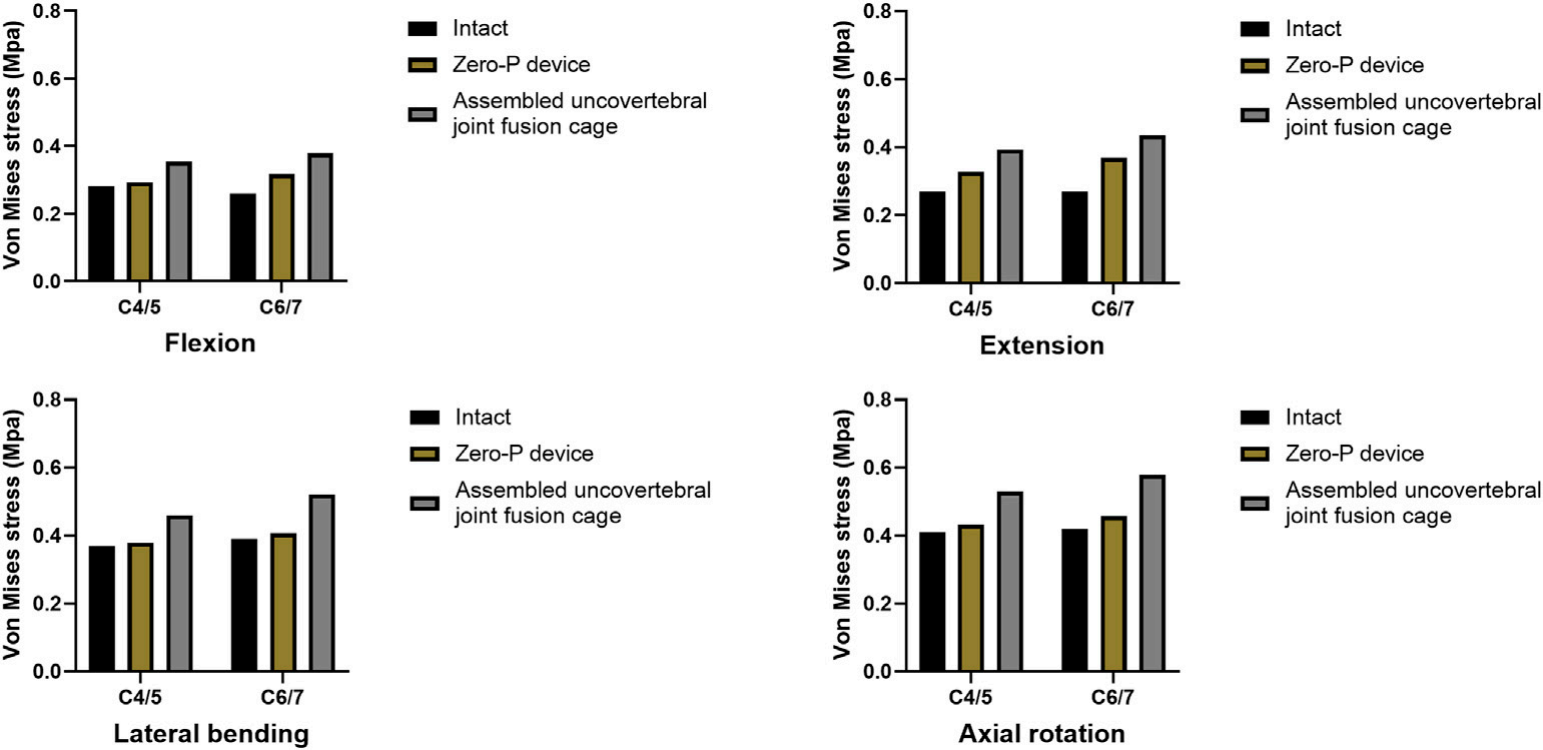
Intradiscal pressure in adjacent levels (the intact model *vs*. Zero-P device *vs*. the assembled uncovertebral joint fusion cage).

**FIGURE 6:**
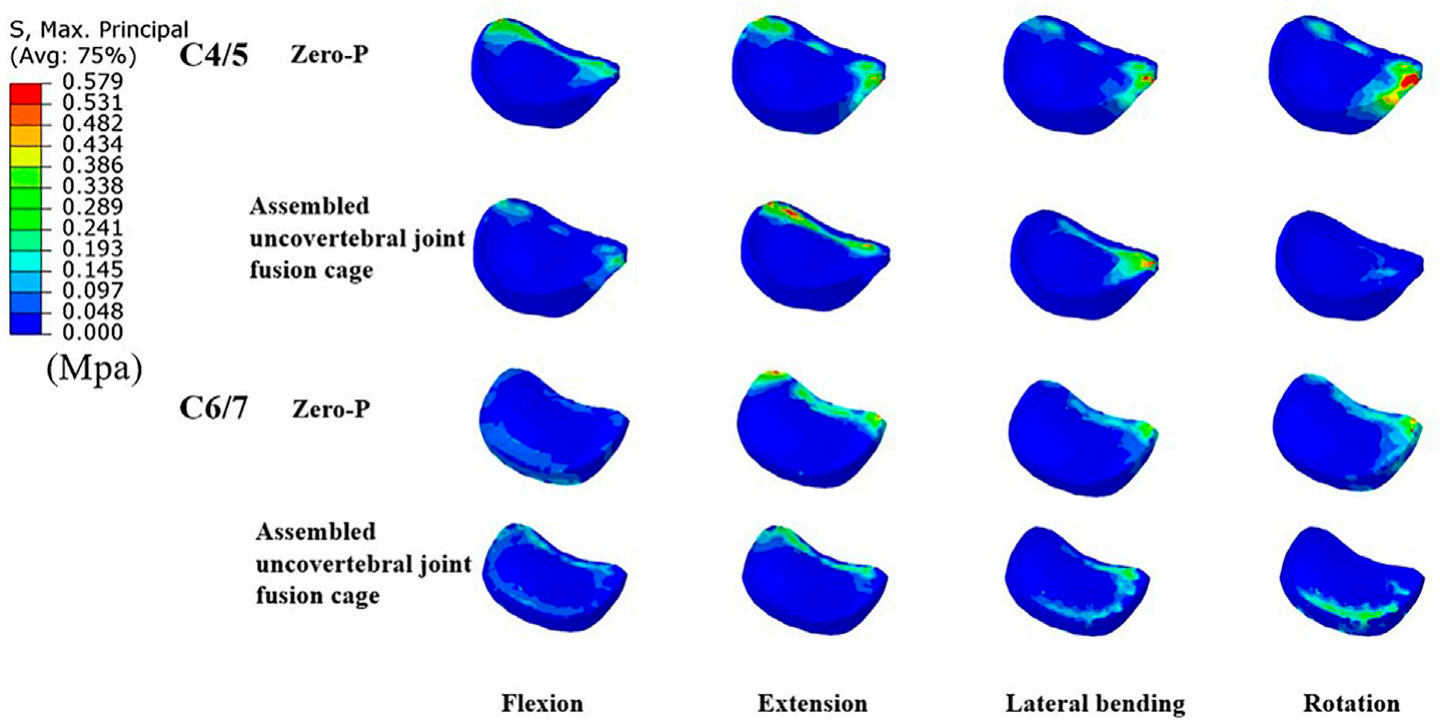
Cloud map of maximum Intradiscal pressure in adjacent levels (the comparisions between Zero-P device and the assembled uncovertebral joint fusion cage during flexion, extension, lateral bending and rotation).

Intradiscal pressure (IDP) measures at C2/3, C3/4, superior adjacent (C4/5), and inferior adjacent (C6/7) segments are shown in Figure 5. As expected, the intradiscal pressure at the adjacent levels in both groups was increased when compared with the intact model. The maximum increase of IDP measures was noted at the inferior adjacent (C6/7) segments under all motions in both models. Notably, the IDP measures in the adjacent segments of the assembled uncovertebral joint fusion cage model were comparable to that in the Zero-P group, only 0.061–0.121 Mpa higher (Figure 5). Stress contour diagrams in all movements in both the assembled uncovertebral joint fusion cage and the Zero-P device group are shown in Figure 6.

### Maximum stress in the end plate–cage interface at the treatment level

**FIGURE 7:**
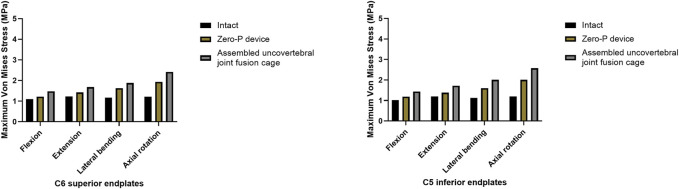
Maximum stress in the cage-endplate interface at surgical levels (the intact model *vs*. Zero-P device *vs*. the assembled uncovertebral joint fusion cage).

A comparison of the maximum von Mises stress developed on the cage–end plate interfaces is shown in Figure 6. The maximum stress on the surface of the C6 superior and C5 inferior end plates of both surgical groups was compared with the intact model. Under flexion, extension, lateral bending, and axial rotation movements, the maximum stress in the C5 inferior end plate of the assembled uncovertebral joint fusion cage group was 1.435, 1.721, 2.009, and 2.575 Mpa, respectively, which is slightly higher than in the Zero-P group (1.189, 1.383, 1.598, and 2.011 Mpa, respectively). Under flexion, extension, lateral bending, and axial rotation, the maximum stress in the C6 superior end plate of the assembled uncovertebral joint fusion cage group was 1.479, 1.680, 1.880, and 2.413 Mpa, respectively, which is slightly higher than in the Zero-P group (1.220, 1.423, 1.633, and 1.936 Mpa, respectively) (Figure 7).

### Maximum stress of the fusion cage

**FIGURE 8:**
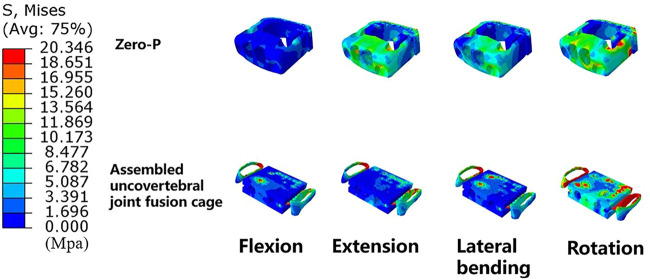
Cloud map of maximum stress of the fusion cage (Zero-P device *vs*. the assembled uncovertebral joint fusion cage).

Stress contour diagrams in all movements in both surgical groups are shown in Figure 7. In the assembled uncovertebral joint fusion cage group, the stress was mainly concentrated in the wings on both sides, reaching 13.4–20.4 Mpa, whereas in the Zero-P group, it was concentrated in the anterior region of the Zero-P device, reaching 11.1–17.4 Mpa (Figure 8).

## Discussion

This study assessed the biomechanical performance of the assembled uncovertebral joint fusion cage in single-level ACDF, which was then compared with that of Zero-P in terms of the range of motion, FCF, IDP, and stress in the end plate–cage interface and also explored the underlying mechanism by which the uncovertebral joint fusion accelerated bone fusion.

### Construct stability

The results showed that the ROMs of the surgical segments in all movement directions decreased by 85.9%–91.6% postoperatively, which is consistent with the outcomes of previous literature (Hua et al., 2020b; Ke et al., 2021). Attributed to the anterior fixation and interbody fusion, sacrificed ROM at the fused segments mainly indicated strong immobilization (Gao et al., 2012; Song and Choi, 2014). Stulik et al. (2007) found that relatively loose internal fixation would lead to excessive relative movement between the bone graft and bone graft bed, easily resulting in the formation of pseudarthrosis. Vandamme et al. (2007) proposed that micromotion controlled within a certain small range can promote bone formation and the occurrence of bone integration. As shown in the results, slightly smaller ROMs of the fused segment in the assembled uncovertebral joint fusion cage group were achieved when compared with the Zero-P group, suggesting that the assembled uncovertebral joint fusion cage could provide strong immobilization, similar to the Zero-P device. Since the uncinate cage increased intervertebral stability at an early stage and limited excessive mobility, it might have effectively promoted the fusion of more parts of the intervertebral space, thus providing the basis for the assembled uncovertebral joint fusion cage with bone grafting in the inter–end plate and bilateral uncovertebral joint to promote bone fusion (Vandamme et al., 2007).

Due to the progressive enhancement of bony fusion at the inter–end plate and bilateral uncovertebral joint, the stiffness of the anterior column increased, which further improved the stability of the construct (Zhang et al., 2022b). It is worth noting that the ROMs of the unfused levels in all models increased by 10.4%–73.2% for all motions when compared to the intact model. The increased mobility of the unfused segment when compared to that of the normal group might be due to the loss of fusion of the fused segments and the compensation of the preserved normal cervical mobility. ACDF was of sacrificed ROM at the fused segments, while increased stress in the adjacent segment may be an important cause for developing adjacent segment degeneration (ASD) (Eck et al., 2002; Hua et al., 2020a; Ke et al., 2021). Considering that the ROM in the assembled uncovertebral joint fusion cage model achieved similar results to those after implantation with the Zero-P device, it is prudent to speculate that the assembled uncovertebral joint fusion cage did not significantly accelerate the degeneration of adjacent segments when compared with Zero-P. In general, the assembled uncovertebral joint fusion cage not only improves stability immediately after surgery but also has great potential for maintaining long-term stability after the operation.

### Risks of degeneration at adjacent segments

Facet contact force (FCF) tended to decrease at the fused segments in both groups due to rigid fixation without relative motion. Under the extension, all unfused levels exhibited a substantial increase in FCF, which was consistent with the change in ROMs. Though stable segmental fixation is necessary for bony fusion, a stiff segment may result in increased FCF and IDP at adjacent levels, thus contributing to adjacent segments degeneration (Hilibrand and Robbins, 2004; Dickerman et al., 2009; Park et al., 2014; Kim et al., 2015; Hashimoto et al., 2019). Promisingly, the FCF increase inside the adjacent facet joints was lower in the assembled uncovertebral joint fusion cage group than in the Zero-P device group, indicating that the assembled uncovertebral joint fusion cage, to a certain degree, may have prevented the development of ASD. However, we noticed that the IDP at the adjacent levels in the assembled uncovertebral joint fusion cage group was greater than in the Zero-P device group, which might have been due to stronger fixation by the surgical segment and faster bone osseointegration after surgery provided in the assembled uncovertebral joint fusion cage when compared with the Zero-P device. Nevertheless, the IDP measures in the assembled uncovertebral joint fusion cage model were comparable to those in the Zero-P group and were only higher by 0.061–0.121 Mpa.

It could be seen from the intradiscal pressure nephograms that the stress distribution with the Zero-P device in ACDF was mainly concentrated at the margin of the intervertebral disc. Although disc stress in the assembled uncovertebral joint fusion cage group was slightly higher than that in the Zero-P group, no such degree of stress concentration was observed, suggesting that comparing only maximum stress values might not be an accurate assessment of the biomechanical disc stress changes in the adjacent segments (Figure 6). Thus, we could cautiously infer that the assembled uncovertebral joint fusion cage did not significantly affect the biomechanics of the adjacent segments when compared with the Zero-P device, but this should be confirmed in long-term follow-up. Additionally, the material properties of intervertebral discs in the FE models may influence the biomechanical results. Nishida et al. (2022), Nikkhoo et al. (2019), Gandhi et al. (2019), and Chen et al. (2022) developed an intervertebral disc model with hyperelastic material properties. Also, a lumbar disc geometry and the properties of disc annulus fibrosis described using a microstructure-based chemo-viscoelastic model have been constructed in some studies (Kandil et al., 2019; Khalaf and Nikkhoo, 2021). Ebrahimkhani et al. (2022) exploited a novel musculoskeletal finite element (MS-FE) spine model in which the intervertebral discs with the nucleus and annulus as a composite of the homogeneous matrix reinforced by collagen fiber networks was constructed. Therefore, it might be more realistic to analyze the biomechanics of adjacent discs through advanced models.

### Cage subsidence

The concentration of high stress at the end plate–cage interface plays an important role in facilitating the penetration of the cage into the end plate and inducing cage subsidence (Chen et al., 2008; Lu et al., 2017). In this present study, end plate stress of both surgical groups was greater than that in the intact model. The outcomes of end plate stress show that the assembled uncovertebral joint fusion cage could still achieve promising results when compared with ACDF using the Zero-P device, though it was 0.246–0.560 Mpa higher. However, cage subsidence is a relatively uncommon postoperative complication in ACDF. Recent studies have demonstrated that ACDF using a Zero-P implant provides satisfactory clinical efficacy and acceptable safety, with a cage subsidence of 7.4%–13.58% at last follow-up (Li et al., 2017; Shen et al., 2018; Sun et al., 2020; Abudouaini et al., 2021b). Although subsidence of the cage had little influence on the clinical outcome in most patients, kyphosis, neurological deterioration, and instrumental complications might occur in some severe cases (Chen et al., 2016; Noordhoek et al., 2018; Oliver et al., 2018). Sun et al. (2021) determined the average stress of the end plate–cage interface from 0.9420 Mpa to 2.0423 Mpa in various fusion cages. Li et al. (2020) found that end plate stress peaks were the highest with the cage plate (0.6–2.4 MPa), followed by ACDF with the Zero-P device (0.5–2.3 MPa). Considering that end plate stress after the assembled uncovertebral joint fusion cage implantation was 1.435–2.575 Mpa, which is consistent with the abovementioned studies, it also supports that the assembled uncovertebral joint fusion cage may not increase the risk of end plate failure when compared with the Zero-P device.

In addition, great attention should be paid to the maximum stress that the end plate can withstand. Kwon et al. (2016) determined that the maximum peak loads at end plate failure for static and expandable spacers were 1764 N (±966 N) and 2284 N (±949 N), respectively. Zhang et al. (2008) determined that the average end plate failure load was 1,875 ± 1,023 N. Based on the abovementioned reference data, we calculated the stress threshold of the end plate, which was ∼25.8 Mpa when the cage–end plate contact area was 31 mm^2^. In addition, the assembled uncovertebral joint fusion cage seemed safe and may not cause end plate failure because the maximum stress at the end plate–cage interface was no more than 3 Mpa. However, current methods for making reasonable estimates of stress thresholds for subsidence are still limited and not individualized. Because the physiological curvature of the cervical spine is anteriorly convex and loads on the anterior spine are eccentric relative to the posterior spine (Zhang et al., 2022b), the failure loads were related to many factors, such as the applied high tensile strains (Fields et al., 2010), bone–end plate contact area or cage shape (Tan et al., 2005), and bone mineral density (BMD) or cage placement (Labrom et al., 2005). Lin et al. (2021, 2022) determined subsidence by measuring the distance that penetrated both the cage and screw into the end plate bone. Therefore, it is necessary to adopt a more rational and individual approach in estimating the maximum stress to failure loads, and we aim to perform relevant targeted optimizations in future studies. In our previous goat model research (Shen et al., 2021), no screw loosening, screw breakage, cage displacement, or subsidence was observed for the uncovertebral joint fusion cage during the 6 months of follow-up. In our prospective, randomized, controlled trial study, there were no cage subsidence and displacement, screw loosening, and fracture in the uncovertebral joint fusion cage group during the 6-month follow-up. Both studies indicated the safety and efficacy of the assembled uncovertebral joint fusion cage.

### Osteogenesis promotion

Another important finding was that stress was mainly concentrated in the wings on both sides, reaching 13.4–20.4 Mpa in the assembled uncovertebral joint fusion cage group. It had been stated that if E signifies the typical peak strains of load-bearing bones, then healthy small and large load-bearing bones should satisfy this criterion: 2 MPa < E < 20 MPa (Frost, 2004). As mentioned above, the uncovertebral joint fusion cage effectively achieved early bone formation and fusion, probably due to proper distribution of stress concentration on both sides of the wings. This finding was clinically important because the bone graft area is located in the uncovertebral joint area on both sides of the assembled uncovertebral joint cage, which might receive proper stress stimulation to promote osteogenesis. However, simulation of the “sufficient osteointegration” stage was required. Our results have suggested greater stress in the uncovertebral joint area, which could also explain the increased speed and quality of bone healing with bone grafting in the uncovertebral joint area during the short term. The method of immediate postoperative testing of biomechanical properties was adopted in most studies (Wu et al., 2019; Huang et al., 2022; Liu et al., 2022; Lv et al., 2022; Zhang et al., 2022a). Considering that osseointegration is a long-term process, a longer dynamic observation of the stress distribution in the region of the uncovertebral joint is necessary.

FE analysis is a valuable method to predict trends after implantation in different cages, providing certain guidance for treatment. However, several limitations of the present study should be discussed. First, the FE model of the cervical spine was simplified to improve the efficiency of convergence in the FE study, which may not simulate the actual biomechanical environment, particularly for end plates at the implanted levels. Second, the FE model was constructed with a healthy cervical spine rather than a degenerative one, and only C5/6 (the most commonly involved one-level ACDF) was implanted for analysis. Thus, the model may not perfectly represent real-world clinical scenarios. However, the current study aimed to provide a trend instead of natural status. Several recent studies have adopted similar methods in developing the finite element spine model and reported acceptable results (Wu et al., 2019; Liang et al., 2022; Zhang et al., 2022b). Third, the geometry of the human spine varies among individuals, while the model of this present study was developed on the basis of the data from a single patient. Thus, the current modeling data should be interpreted with caution. Fourth, this study simplifies the musculoskeletal system, and the results under this ideal condition did not fully reflect the actual postoperative situation. Although this study aimed to provide a biomechanical reference for clinics, the manipulation of the muscular–ligamentous system in this finite analysis was based on previous literature (Fu et al., 2022; Huang et al., 2022; Shen et al., 2022; Zhang et al., 2022b). Last, linear elastic materials were adopted for the cervical vertebral body and intervertebral disc. In our study, the ligamentous complex, which includes anterior longitudinal ligaments (ALL), posterior longitudinal ligaments (PLL), capsular ligament (CL), ligamentum flavum (LF), interspinous ligaments (IL), and supraspinous ligament (SL), was established using non-linear tension-only truss elements. The facet joint was built as a non-linear three-dimensional contact problem using surface-to-surface contact elements. A face-to-face contact algorithm was used to define facet joint interaction, which was assumed with frictionless sliding contact. Also, we chose linear elastic materials for the vertebral body and intervertebral disc for good convergence in the calculating process. Although linear elastic materials may partially affect the biomechanical environment, the present study primarily focused on the changing trends following the implantation of a newly assembled uncovered vertebral joint fusion cage. The material properties should be re-assessed if the objective of the study changes. Analogously, several recent studies have adopted similar methods for developing a finite element cervical spine model and provided satisfactory results (He et al., 2021; Hua et al., 2020a; Hua et al., 2020b; Lin et al., 2021; Wo et al., 2021; Shen et al., 2022; Zhang et al., 2022b). Given that material properties such as hyperelastic, viscoelastic, or poroelastic materials within the intervertebral disc can result in better biomechanical predictions, a more realistic model has to be developed in future studies.

## Conclusion

In conclusion, this study showed that the assembled uncovertebral joint fusion cage provided strong immobilization similar to the Zero-P device. Compared with the Zero-P group, the assembled uncovertebral joint fusion cage achieved similar resultant values regarding FCF, IDP, and stress in the end plate–cage interface. Altogether, the assembled uncovertebral joint fusion cage was non-inferior to the Zero-P device in terms of biomechanical properties. Moreover, the proper distribution of stress concentration in the wings on both sides may play an important role in achieving early bone formation and fusion of the assembled uncovertebral joint fusion cage.

## Data Availability

The original contributions presented in the study are included in the article/Supplementary Material; further inquiries can be directed to the corresponding author.

## Abbreviations

ACDF, anterior cervical discectomy and fusion; ALL, anterior longitudinal ligament; FCF, facet contact force; FE, finite element; IDP, intradiscal pressure; PLL, posterior longitudinal ligament; ROM, range of motion.
